# Predictors of white matter hyperintensities in the elderly Congolese population

**DOI:** 10.3389/fnagi.2025.1491477

**Published:** 2025-04-08

**Authors:** Emile Omba Yohe, Alvaro Alonso, Daniel L. Drane, Saranya Sundaram Patel, Megan Schwinne, Emmanuel Epenge, Guy Gikelekele, Esambo Herve, Immaculee Kavugho, Nathan Tshengele, Samuel Mampunza, Lelo Mananga, Liping Zhao, Deqiang Qiu, Anthony Stringer, Amit M. Saindane, Jean N. Ikanga

**Affiliations:** ^1^Department of Epidemiology, Rollins School of Public Health, Emory University, Atlanta, GA, United States; ^2^Departments of Neurology and Pediatrics, Emory University School of Medicine, Atlanta, GA, United States; ^3^Department of Rehabilitation Medicine, Emory University School of Medicine, Atlanta, GA, United States; ^4^OneRehab, Dallas, TX, United States; ^5^Department of Biomedical Informatics, School of Medicine, Emory University, Atlanta, GA, United States; ^6^Department of Neurology, University of Kinshasa, Kinshasa, Democratic Republic of Congo; ^7^Department of Psychiatry, School of Medicine, University of Kinshasa and Catholic University of Congo, Kinshasa, Democratic Republic of Congo; ^8^Memory Clinic of Kinshasa, Kinshasa, Democratic Republic of Congo; ^9^Department of Biostatistics and Bioinformatics, Rollins School of Public Health, Emory University, Atlanta, GA, United States; ^10^Department of Radiology and Imaging Sciences & Department of Biomedical Engineering, School of Medicine, Emory University, Atlanta, GA, United States; ^11^Departments of Radiology and Imaging Sciences and Neurosurgery, Emory University School of Medicine, Atlanta, GA, United States

**Keywords:** white matter hyperintensities, white matter disease, dementia, cardiovascular risk factors, Congo, sub-Saharan Africa, cerebral small vessel disease, cerebrovascular disease

## Abstract

**Introduction:**

White matter hyperintensities (WMHs) are strongly linked to cardiovascular risk factors and other health conditions such as Alzheimer’s disease. However, there is a dearth of research on this topic in low-income countries and underserved populations, especially in the Democratic Republic of Congo (DRC) where the population is aging rapidly with increasing cardiovascular risk factors and dementia-related diseases. This study evaluates health factors associated with WMH in the elderly Sub-Saharan Africa (SSA), specifically Congolese adults.

**Methods:**

In a cross-sectional study of 77 people from the DRC, participants underwent neuroimaging to analyze WMHs volume and completed clinical evaluation, laboratory-based blood exams, self-reported questionnaires, and interviews. A simple linear regression model was conducted to test the association between WMHs and potential predictors (dementia, age, sex, hypertension, diabetes, tobacco abuse, stroke, high cholesterol, cardiovascular medication, and alcohol abuse). Stepwise selection and backward elimination analyses were performed to obtain the final model. Finally, a multiple linear regression model was conducted to assess the association between WMHs and variables retained in the final model (dementia, sex, and age).

**Results:**

Of the 77 individuals, 47 (61%) had dementia, 40 (52.6%) were males, and the mean age was 73 years (± 8.0 years standard deviation). In simple linear regression models, WMHs was significantly associated with dementia (expβ1 = 1.75, 95% CI = 1.14–2.71, *p*-value = 0.01) though it had a weak association with age (expβ1 = 1.03, 95% CI = 1.00–1.05, *p*-value = 0.05) and sex (male) (expβ1 = 0.66, 95% CI = 0.43–1.01, *p*-value = 0.05). In multiple linear regression models, WMHs was statistically significantly associated with dementia (expβ1 = 1.97, 95% CI = 1.31–2.95, *p*-value =0.001), male sex (expβ2 = 0.54, 95% CI = 0.36–0.80, *p*-value = 0.003), and age (expβ3 = 1.03, 95% CI = 1.00–1.06, *p*-value = 0.03). However, WMHs was not significantly associated with common cardiovascular risk factors, such as high blood pressure, diabetes, tobacco use, obesity, and high cholesterol levels.

**Discussion:**

WMHs is significantly associated with dementia, sex, and age in the Congolese population. Understanding these predictors may improve our ability to diagnose, assess, and develop preventative treatments for white matter disease in SSA/DRC populations, where neuroimaging is difficult to obtain.

## 1 Introduction

White matter hyperintensities (WMHs), or lesions, are a marker of change to the connective nerve fibers of the brain, potentially contributing to cognitive decline, balance, and mobility issues (Moroni et al., 2020; Karvelas and Elahi, 2023). WMHs are present in approximately 20–50% of the general population in midlife, increasing to more than 90% with advanced age (Karvelas and Elahi, 2023; Roseborough et al., 2023) and can be related to normal aging changes or neurodegenerative diseases, such as Alzheimer’s disease (AD) (Tubi et al., 2020; GBD 2016 Dementia Collaborators, 2019). AD is the leading cause of dementia in individuals aged 65 or older, with vascular dementia being the second most common (Alzheimer's Association Report, 2022; Aarsland, 2020). AD is a significant global burden present in approximately 40–50 million people and it is predicted to be over 150 million by 2050 (GBD 2016 Dementia Collaborators, 2019; Tahami Monfared et al., 2022; Breijyeh et al., 2020). WMHs are found in more than 89% of patients with AD and are typically more severe than WMHs seen in older adults without dementia (Breijyeh et al., 2020; Agosta et al., 2011; Mcaleese et al., 2017). The observed patterns of WMHs can reflect the advanced phase of the disease, as significant and anatomically congruent correlations between WMHs and regional gray matter atrophy have been seen in patients with AD (Agosta et al., 2011).

In vascular dementia (Karvelas and Elahi, 2023), research has found that WMHs are strongly linked to cardiovascular disease (CVD) risk factors (e.g., smoking, hypertension, diabetes, dyslipidemia, physical inactivity, being overweight, and obesity) as a consequence of cerebral small-vessel disease (cSVD) (Teo and Rafiq, 2021; Tsao et al., 2023; Poels et al., 2012). Furthermore, researchers have demonstrated that the association between WMHs and early signs of cognitive decline is detectable many years before the emergence of clinical symptoms of AD and related dementia (ADRD); therefore, WMHs can be used as a surrogate biomarker of cognitive decline and risk of dementia (Elliott et al., 2019). Interestingly, the prevalence of WMHs is greater in the female sex, contributing to a higher incidence and prevalence of dementia in women than in men in older adults (De Leeuw et al., 2001).

There has been substantial research documenting the association between cardiovascular (CV) risk factors, AD, and WMHs in high-income countries (HIC) and populations of European ancestry. However, few studies have investigated the increasing risk of WMHs in low- and middle-income countries (LMIC) (Samba et al., 2016). The predicted increase in dementia-related diseases like WMHs is more susceptible in LMIC, especially in East Asia and Sub-Saharan Africa (SSA), where more than 70% of all people with dementia are expected to live by 2040 (Chadli et al., 2018; Guerchet et al., 2010). The Democratic Republic of Congo (DRC) is undergoing the third phase of the epidemiological transition, which is characterized by a shift in the overall burden of morbidity and mortality from infectious diseases to non-communicable diseases and injuries. This includes conditions such as high blood pressure, diabetes, obesity, and Alzheimer’s disease-related dementias (Chadli et al., 2018). However, there is limited research on WMHs in the Democratic Republic of Congo (DRC) where the population is aging rapidly with high prevalence rates of cardiovascular risk factors and suspected ADRD. When considering the Congolese population, public health authorities should be cautious about directly applying research findings from high-income countries (HIC) and populations of European ancestry, as the prevalence of WMHs and the distribution of WMH-associated risk factors may differ significantly from those observed in HIC populations.

Thus, further research is needed in the DRC to understand health factors associated with WMHs to determine whether age, CV risk factors, and ADRD are associated with WMHs in this population. As WMHs are slowly progressive, early detection will provide the opportunity for early intervention to reduce or delay the occurrence of dementia-related outcomes, such as memory problems, balance issues, and mood changes in the population (Karvelas and Elahi, 2023; Elliott et al., 2019). This study evaluates factors associated with WMHs in a cohort from the elderly Congolese population. We hypothesized that WMHs will be associated with AD-related dementia, CV risk factors and disease, and age.

## 2 Materials and methods

### 2.1 Study design and population

**Figure 1:**
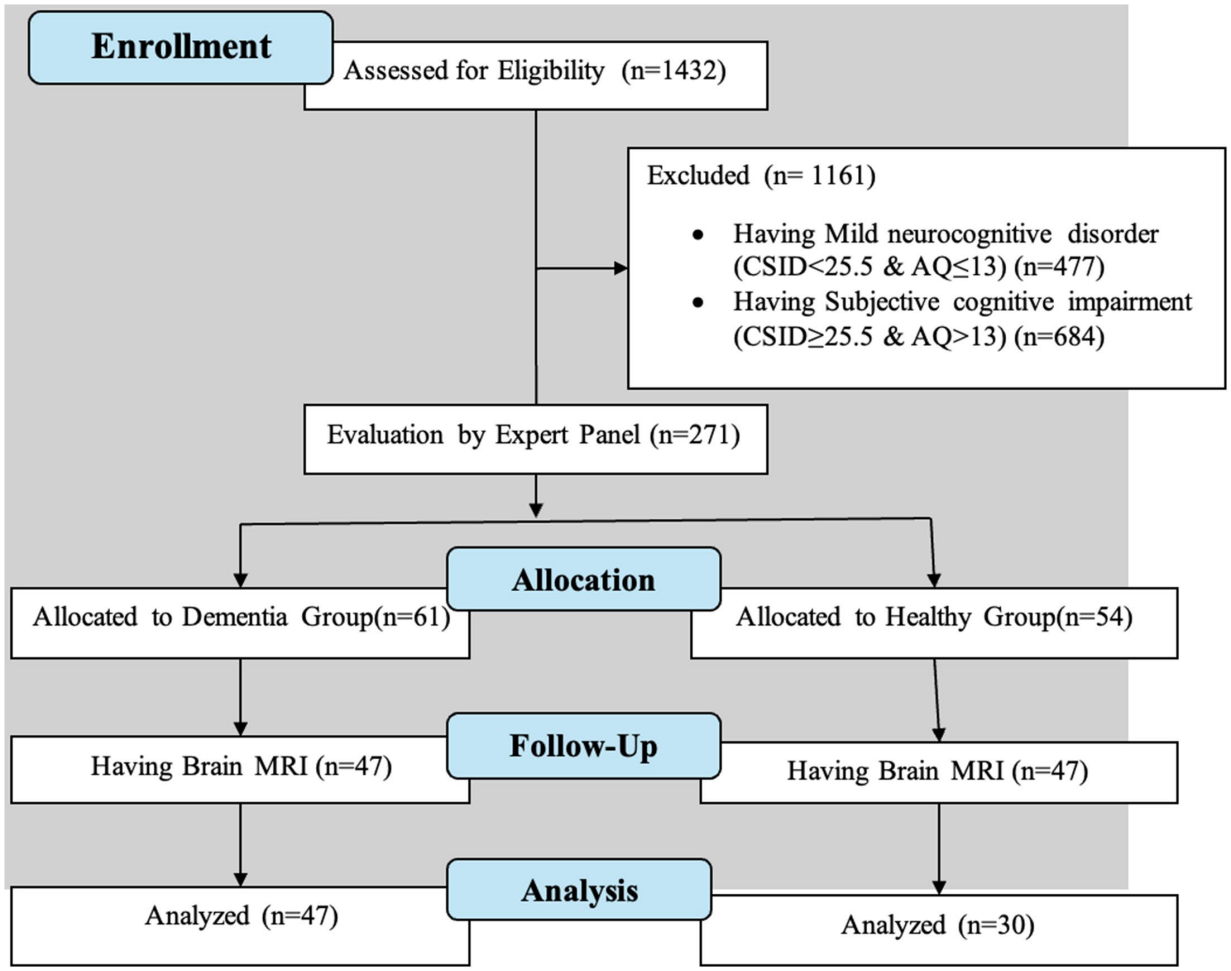
Flow diagram of participant recruitment.

This cross-sectional study was conducted from 2019 to 2022 in Kinshasa, DRC. Initially, people were selected through a community-based recruitment procedure from door-to-door in the city, clinics, hospitals, churches, and older adult associations. Inclusion criteria included 50 years of age or older, diagnosis of major neurocognitive disorder or normal cognition according to DSM-5, having a collateral informant, and being fluent in French or Lingala. Participants were excluded from this study if they had a history of schizophrenia, neurological, or other medical conditions potentially affecting the central nervous system (CNS), such as brain tumors or congenital brain defects. Due to the lack of clear cutoff values for AD biomarkers in the SSA to clinically confirm the diagnosis of probable AD, we used two screening measures with high sensitivity and specificity for identifying individuals with dementia in Western cohorts, the Community Screening Instruments for Dementia (CSID) and Alzheimer’s Questionnaire (AQ) (Schwinne et al., 2024; Ikanga et al., 2023). The AQ distinguishes between those with AD from healthy controls. The CSID Questionnaire has been extensively used in many international and SSA dementia studies, including studies in Nigeria, Uganda, and South Africa (Ikanga et al., 2023). Based on cognitive and functional deficits for Diagnostic and Statistical Manual of Mental Disorders, Fifth Edition, Text Revision (DSM-5-TR) diagnostic criteria, we used Brazzaville cut-offs of CSID, the closest city from Kinshasa, to classify participants. Similar to our prior study, participants were classified using CSID and AQ scores (Figure 1), which yielded 4 groups: major neurocognitive disorder/dementia, mild neurocognitive disorder (MND), subjective cognitive impairment, and healthy control (HC), i.e., normal cognition. For the AQ, only the total score was used, based on 27 possible points and a cutoff score of 13 or more points suggestive of dementia. Written informed consent was obtained from all participants before undergoing any study procedures approved by the Ethical Committee and Institutional Review Boards of the University of Kinshasa. All participants were financially compensated for their time (Schwinne et al., 2024).

A panel consisting of a neurologist, psychiatrist, and neuropsychologist reviewed screening tests, clinical interviews, and neurological examination of subjects. Sixty-one individuals were confirmed with a diagnosis of major neurocognitive disorders and 54 were deemed HCs. HCs were matched based on age, sex, and education levels before the MRI procedure. Brain MRI was then performed, resulting in the final sample of 77 subjects, 47 with dementia and 30 HCs.

### 2.2 Measures

Demographic, socioeconomic, and medical history (e.g., stroke, tobacco use, alcohol use, and cardiovascular medication) information were obtained through participants’ self-reported questionnaires and interviews. Participants were categorized into four different age groups: 50–64, 65–74, 75–84, and 85+ and four different education groups: primary school (1–6 years), secondary school (7–12 years), some or completion of university (13–17 years), and beyond university (18+ years) (Ikanga et al., 2023).

Three blood pressure measurements were conducted by a medical resident at complete rest. A participant was considered to have hypertension if their systolic blood pressure was greater than 139 mmHg and diastolic was greater than 89 mmHg. Participants were considered to have diabetes mellitus type 2 if their blood sugar had an HbA1C level ≥ 6.5% or having a fasting blood glucose ≥7.0 mmoL/L. The cholesterol level was detected by blood test. Participants were considered to have high cholesterol if their total cholesterol is above 200 mg/dL.

### 2.3 Neuroimaging parameters

Each participant was scanned using a 1.5 Tesla MRI unit scanner (Siemens, Magneton Sonata) at HJ Hospitals in Kinshasa, DRC, using the same standardized imaging acquisition protocol based on the Alzheimer’s Disease Research Center (ADRC) protocol of Emory University (Ikanga et al., 2023). It consisted of sagittal volumetric T1-weighted (MP-RAGE), coronal T2-weighted, axial diffusion-weighted, T2-weighted, and T2-FLAIRE sequences. High-resolution structural images were obtained using a T1-weighted MP-RAGE Sequence with the following parameters: Repetition Time (TR) = 2,200 ms; minimum full Echo time (TE) = 1,000 ms; Flip angle = 8^0^; Field of view (FOV) = 250 mm; acquisition matrix = 192 × 184, yielding a voxel size of approximately 1.25 mm × 1.25 mm × 1.2 mm.

### 2.4 Quantification of WMHs

To identify and extract WMHs volume, brain segmentation was performed by experts at Emory University. To obtain a white matter hyperintensities (WMHs) volume on an MRI scan, a Fluid Attenuated Inversion Recovery (FLAIR) sequence was used to identify and segment the areas of hyperintense white matter on the image. Then, the volume of those selected voxels was calculated by measuring the bright spots that represent damaged white matter within the brain. The quantitative volumetric analysis, visualization, and processing were conducted using FreeSurfer, which enabled us to label both the cortical (parcellation) and subcortical (segmentation) structures. An experienced subspecialty-certified neuroradiologist reviewed images to ensure that automated processes resulted in accurate data (Ikanga et al., 2023). The number of chronic brain parenchymal micro hemorrhages was recorded, and the lobar volume loss pattern of the brain was assessed. Regional brain volume for both cortical and sub-cortical brain regions was calculated. Variations in head size among individuals were controlled for by adjusting for total intracranial volume in the subsequent statistical analysis. Finally, the presence or absence of any additional abnormalities was noted, and participants with neuroimaging evidence indicated an etiology other than probable AD (e.g., the presence of a brain tumor), were excluded from the analysis.

The Fazekas scale for white matter lesions was used to quantify the amount of white matter T2 hyperintense lesions, typically attributed to chronic small vessel ischemia (Ikanga et al., 2023) which is mainly used to describe white matter disease severity. It divides the white matter into periventricular and deep white matter. Each region is graded based on the size and confluence of lesions. For this analysis, we used the deep white matter (DWM) classification, which is crucial in assessing white matter disease due to a chronic small vessel ischemia, patients with possible dementia, and it is the component that is usually reported in clinical research (e.g., Fazekas grade 2) (Ikanga et al., 2023).

- 0 = absent
- 1 = punctate foci
- 2 = beginning confluence
- 3 = large confluence areas.

### 2.5 Statistical analyses

All statistical analyses were completed using SAS version 9.4 statistical software. Descriptive statistics (e.g., frequencies, percentages, means, and standard errors) were generated for both the overall sample and between dementia (Table 1). WMHs volumes were log-transformed for normality. A univariable analysis in a simple linear regression model was conducted to test the association between WMHs (dependent variable) and each predictor (primary independent variable). Dementia, age, age decade, sex, hypertension, diabetes, tobacco abuse, stroke, high cholesterol, cardiovascular medication, and alcohol abuse predictors were considered predictors. A multivariable analysis in a multiple linear regression model was conducted to assess the association of WMHs and all the predictors retained in the final model. Multivariable analysis started with a stepwise selection using a *p*-value cut-off of 0.20 for variable entry and removal. Given our small sample size, using a higher *p*-value cutoff of 0.20 is essential to prevent the exclusion of potentially important variables that may not show statistical significance due to low statistical power. Then, backward elimination was performed to remove insignificant variables at the 0.05 level to obtain the final model. The predictors that were retained in the final model were dementia, sex, and age. The results were expressed as exp. (beta) coefficients with corresponding 95% confidence intervals. All statistical tests were two-sided; *p*-values <0.05 were considered statistically significant. The Bonferroni method was applied in the final model to adjust for multiple comparisons. The threshold for statistical significance was set at *α* = 0.05/3, which equals α = 0.016. Therefore, WMHs volumes were considered significant only if the associated *p*-values from these tests were less than 0.016.

**Table 1:** Descriptive characteristics of the sample population, stratified by dementia.

Variables, *n* (%) or mean (SD)	HC (*n* = 30)	Dementia (*n* = 47)	Overall (*n* = 77)
Demographics			
Male	18 (62.1%)	22 (46.8%)	40 (52.6%)
Body Mass Index, kg/m^2^^a^	19.4 (9.9)	20.7 (9.04)	20.2 (9.4)
Age, years	71.6 (8.6)	73.04 (7.7)	72.5 (8.04)
Age groups, years^b^			
50–64	3 (10.3%)	4 (8.5%)	7 (9.2%)
65–74	9 (31%)	11 (23.4%)	20 (26.3%)
75–84	13 (44.8%)	23 (48.9%)	36 (47.4%)
85+	4 (13.8%)	9 (19.2%)	13 (17.1%)
Years of education^a^	10.7 (5.2)	8.2 (5.3)	9.13 (5.4)
Education levels, years^b^			
Primary school (1–6)	1 (3.5%)	6 (12.8%)	7 (9.2%)
Secondary school (7–12)	5 (17.2%)	14 (29.8%)	19 (25%)
Some/completed university (13–17)	13 (44.8%)	17 (36.2%)	30 (39.5%)
Beyond University (18+)	10 (34.5%)	10 (21.3%)	20 (26.3%)
WMH volume, mm^3^^a^	9,087 (9830)	15,744 (15467)	13,151 (13869)
WMH-Fazekas^b^			
Absent	5 (18.5%)	0 (0.00%)	5 (6.8%)
Punctuate foci	13 (48.2%)	25 (54.4%)	38 (52.1%)
Beginning confluence	8 (29.6%)	9 (19.6%)	17 (23.3%)
Large confluence areas	1 (3.7%)	12 (26.1%)	13 (17.8%)
Hypertension	13 (44.8%)	26 (55.3%)	39 (51.3%)
Diabetes	5 (17.2%)	1 (2.1%)	6 (7.8%)
High cholesterol	3 (10.3%)	1 (2.1%)	4 (5.3%)
Stroke	0 (0.0%)	5 (10.6%)	5 (6.6%)
Tobacco abuse	3 (10.3%)	4 (8.5%)	7 (9.21%)
Alcohol abuse	4 (13.8%)	10 (21.3%)	14 (18.4%)
Cardiovascular medication	7 (24.1%)	6 (12.8%)	13 (17.1%)

WMH, While Matter Hyperintensity.

a These variables are reported as mean (SD).

b These values may not sum to the total due to missing data.

## 3 Results

### 3.1 Descriptive characteristics of the sample population

Participants were evenly distributed between males (52.6%) and females (47.4%). The mean age was 73 years and participants’ age groups, education levels, and body mass index showed no significant differences between dementia and control groups. Regarding WMHs, the sample mean WMHs volume was 13,151 mm^3^ [standard deviation (SD) = 13,869 mm^3^], with dementia cases having a higher mean WMH volume (15,745 mm^3^; SD = 15,467 mm^3^) than HC (9,088 mm^3^; SD = 9,830 mm^3^). There was a significant difference in mean WMHs volume between participants with dementia (WMHs = 15745.0, SD = 15467.4) and healthy control (HC) (WMHs = 9087.5, SD = 9830.2); t (75) = −2.10, *p*-value = 0.039. A little more than half of the participants (51.3%) had hypertension, with a higher prevalence among dementia compared to HC cases (55.3% and 44.8%, respectively). Additionally, more dementia cases (11%) reported a history of stroke compared to the healthy control cases (0%).

### 3.2 Association between WMHs and predictors

**Figure 2:**
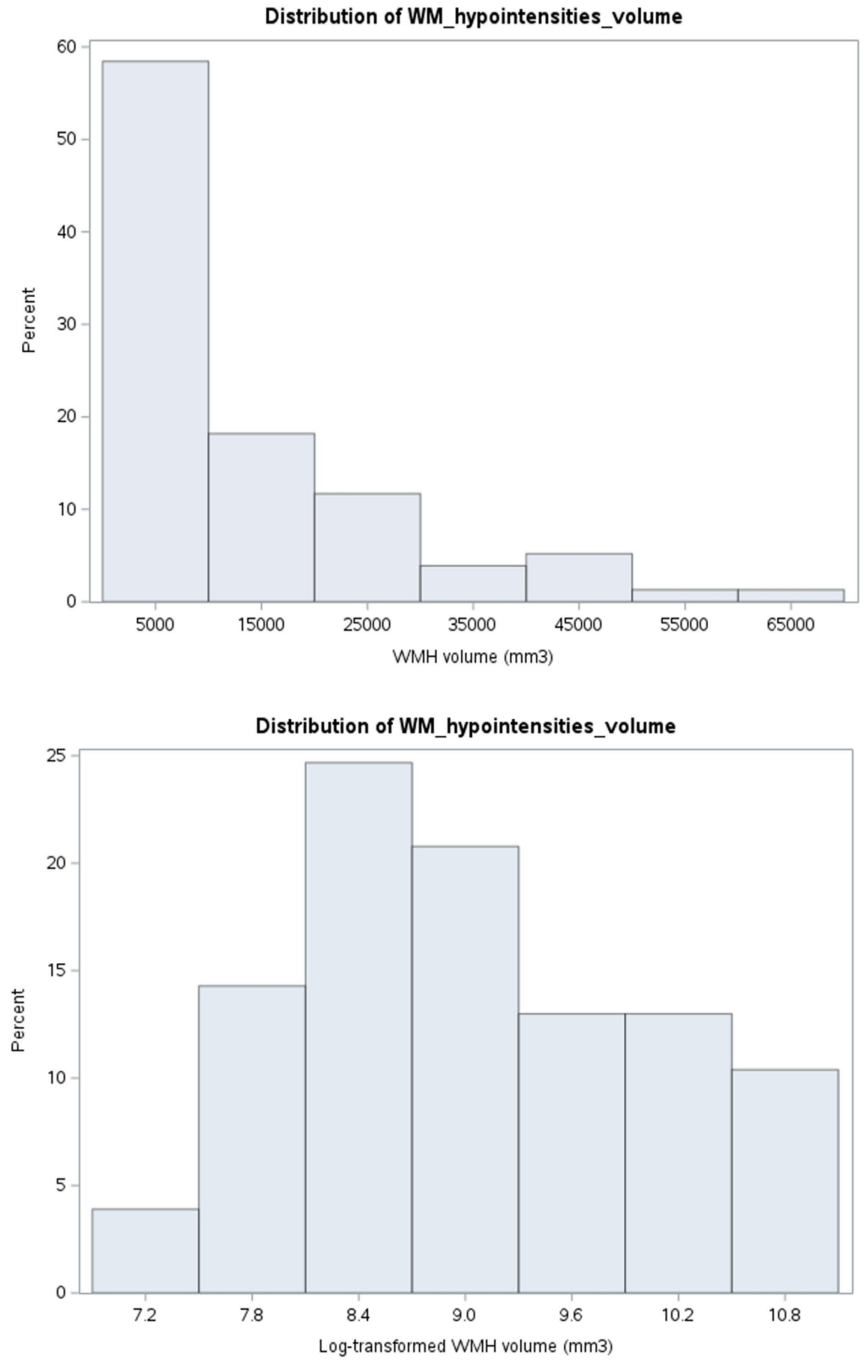
Distribution of white matter hyperintensities.

The association between WMHs and its predictors was performed using the log-transformed value of WMHs volume (Figure 2) in simple and multiple linear regression models.

In a simple linear regression model, WMHs was significantly associated with dementia (expβ_1_ = 1.75, 95% CI = 1.14–2.71, *p*-value = 0.01). Participants with dementia had a 75% increase in WMH volume compared to HC. WMHs had a statistically weak association with age (expβ_1_ = 1.03, 95% CI = 1.00–1.05, *p*-value = 0.05) and sex (male) (expβ_1_ = 0.66, 95% CI = 0.43–1.01, *p*-value = 0.05). Male participants had a WMHs volume 34% lower than female participants, and when participants’ age increases by 1 year, it is associated with a 3% increase in WMHs volume. There were no statistically significant associations between WMHs and other predictors, such as age decade (expβ_1_ = 1.21, 95% CI = 0.94–1.56, *p*-value = 0.12), diabetes (expβ_1_ = 1.84, 95% CI = 0.82–4.10, *p*-value = 0.13), alcohol abuse (expβ_1_ = 0.67, 95% CI = 0.38–1.16, *p*-value = 0.15), tobacco abuse (expβ_1_ = 1.66, 95% CI = 0.78–3.50, *p*-value = 0.18), stroke (expβ_1_ = 0.72, 95% CI = 0.32–1.58, *p*-value = 0.40), high cholesterol (expβ_1_ = 1.42, 95% CI = 0.53–3.79, *p*-value = 0.48), hypertension (expβ_1_ = 0.85, 95% CI = 0.56–1.35, *p*-value = 0.54), or cardiovascular medication (expβ_1_ = 0.93, 95% CI = 0.52–1.67, *p*-value = 0.81) (Table 2).

**Table 2:** Associations between WMHs and predictors.

Variable	Exp(β)	95%CI	*p*-value
Dementia	1.75	1.14–2.71	0.01
Age (total years)^a^	1.03	1.00–1.05	0.05
Age decade	1.21	0.94–1.56	0.14
Sex (male)	0.66	0.43–1.01	0.05
Hypertension	0.87	0.56–1.35	0.54
Diabetes	1.84	0.82–4.10	0.13
Tobacco abuse	1.66	0.78–3.5	0.18
Stroke	0.72	0.32–1.58	0.4
High cholesterol	1.42	0.53–3.79	0.48
Cardiovascular medication	0.93	0.52–1.67	0.81
Alcohol abuse	0.67	0.38–1.16	0.15

WMH, While Matter Hyperintensity.

a These variables are reported as continuous.

Our results are presented as the exponentiated beta value of each predictor considered in a simple linear regression model. The result corresponds to the relative increase or decrease in WMH volume associated with a specific variable.

### 3.3 Association between WMHs and final model variables

The summary of the stepwise selection process showed that four variables were selected: dementia (expβ_1_ = 1.95, 95% CI = 1.30–2.93, *p*-value = 0.001), sex (male) (expβ_2_ = 0.53, 95% CI = 0.36–0.80, *p*-value = 0.003), age (expβ_3_ = 1.03, 95% CI = 1.00–1.05, *p*-value = 0.03), and tobacco abuse (expβ_4_ = 1.59, 95% CI = 0.81–3.12, *p*-value = 0.17) (Table 3).

**Table 3:** Summary of stepwise selection.

Variable	Exp(β)	95%CI	*p*-value
Dementia	1.95	1.30–2.93	0.001
Sex (male)	0.53	0.36–0.80	0.003
Age, per year^a^	1.03	1.00–1.05	0.03
Tobacco abuse	1.59	0.81–3.12	0.17

a These variables are reported as continuous.

Stepwise selection uses a *p*-value cut-off of 0.20 for variable entry and removal. Our results are presented as the exponentiated beta value of each predictor considered in a multiple linear regression model. The result corresponds to the relative increase or decrease in WMH volume associated with a specific variable.

Then, after the backward elimination process, only three variables were retained in our final model: dementia (expβ_1_ = 1.97, 95% CI = 1.31–2.95, *p*-value = 0.001), sex (male) (expβ_2_ = 0.54, 95% CI = 0.36–0.80, *p*-value = 0.003), and age (expβ_3_ = 1.03, 95% CI = 1.00–1.06, *p*-value = 0.03) (Table 4).

**Table 4:** Summary of backward elimination/final model.

Variable	Exp(β)	95%CI	*p*-value
Dementia	1.97	1.31–2.95	0.001
Sex (male)	0.54	0.36–0.80	0.003
Age, per year^a^	1.03	1.00–1.06	0.03

a These variables are reported as continuous.

Backward elimination was performed to remove insignificant variables at the 0.05 level to obtain the final model. Our results are presented as the exponentiated beta value of each predictor considered in the final model of a multiple linear regression model. The result corresponds to the relative increase or decrease in WMH volume associated with a specific variable.

The multivariable analysis showed a statistically significant association between WMHs and all three variables in the final model. Dementia was significantly associated with WMHs when controlling sex and age. Participants with dementia had two times higher (97% increase) volume of WMHs than the HC, controlling for sex and age. Sex (male) was significantly less associated with WMHs, when adjusted for dementia and age. Male participants had a 46% decrease in WMHs volume compared to females, controlling for dementia and age. Age was significantly associated with WMHs, controlling for dementia and sex. The increase in 1 year of age was associated with a 3% increase in participants’ WMHs volume, controlling for dementia and sex (Table 4). When employing the Bonferroni method for multiple comparison correction in the final model, only age was not found to be significantly associated with WMHs, while controlling for dementia and sex [age: exp.(β3) = 1.03, 95% CI = 1.00–1.06, *p*-value = 0.03 at *α* = 0.016].

## 4 Discussion

This study evaluated health factors associated with WMHs in the elderly community-based sample from Kinshasa, DRC. We hypothesized that WMHs will be associated with AD-related dementia, CV risk factors and diseases, and age. For the unadjusted analysis, we found a significant association between WMHs and dementia and a weak association between WMHs and the sex and age of the participants. Participants with dementia had a 75% increase in WMHs volume compared to HC. Male participants had a 34% decrease in WMHs volume than female, and the increase in age by 1 year is associated with a 3% increase in WMHs volume. In the adjusted analysis, dementia status, older age, and female sex were significantly associated with a larger volume of WMHs independently of each other. Participants with dementia had almost twice (97% increase) the volume of WMHs than the HC, controlling for sex and age. Male participants had a 46% decrease in WMHs volume compared to female, when adjusted for dementia and age. The increase in 1 year of age was associated with a 3% increase in participants’ WMHs volume, controlling for dementia and sex. However, after applying the multiple comparison correction, there was no significant difference in the increase of WMHs volume when age increased by 1 year, while controlling for dementia and sex. Our results were meaningfully similar to published studies of populations in HIC, where previous researchers suggest that WMHs are associated with an increased risk of cognitive dysfunction and are found in most patients with AD-related dementia (Mcaleese et al., 2017; Hu et al., 2021).

Additionally, researchers indicate that white matter diseases typically begin at midlife, with their prevalence increasing as individuals age. Over 90% of cases of WMHs are observed in older adults (Karvelas and Elahi, 2023).

Moreover, previous studies showed an association between WMHs and sex, as the female population had a higher incidence and prevalence of white matter diseases than the male population (De Leeuw et al., 2001). These previous results align with our findings that WMHs were significantly associated with dementia, sex, and age of participants both in an univariable and multivariable analysis. Our findings differed from those of some published studies, as we did not find a link between WMHs and cardiovascular risk factors or diseases. This discrepancy may be attributed to our small sample size and the relatively low prevalence of cardiovascular risk factors in our sample.

WMHs are confirmatory imaging diagnosis of changes in the brain through MRI, which most patients are not able to afford the cost in the DRC. Clinically, the presence of WMHs on MRI is associated with cognitive decline, balance issues, and mood change (depression) (Karvelas and Elahi, 2023). Our findings provide valuable scientific evidence that can assist physicians and healthcare professionals in caring for patients experiencing dementia-related issues and gait disturbances resulting from white matter hyperintensities (WMHs), even while they await confirmatory brain MRI results. In the population level, our findings will be essential in implementing public health programs that target individual behavioral change to prevent WMHs and stop white matter diseases from getting worse, which will be relevant in decreasing the burden of dementia (disability and loss of life, social isolation, discrimination, stigmatization, and death) in the Congolese population. The results of our study may contribute to the design of clinical trials for WMHs and Alzheimer’s disease, as well as other related dementias within the Congolese population. Our findings may encourage clinical action. A researcher or primary care physician could use the presence of WMHs in a brain MRI as a reason to refer the patient to a neurologist or neuropsychiatrist. This referral would help in managing risk factors and preventing the burden and progression of WMHs.

White matter disease is common in the general population. They can be caused by numerous factors, such as cardiovascular risks and disease, AD-related dementia, age, and sex. Understanding the association between WMHs and its predictors is crucial in implementing early diagnosis, prevention programs, and treatment. This study found that WMHs are significantly associated with dementia (dementia), sex (females), and age (older adults) in the Congolese population. Knowing these predictors of WMHs in the Congolese population could be a useful prevention tool for white matter disease in SSA countries, especially in DRC, where brain MRI diagnosis is difficult to obtain.

Limitations include a relatively small sample size (77 participants) which can subsequently reduce the power of the study and limited the detection of differences that could have been clinically and significantly relevant to discriminate the two groups. Our small sample may not adequately represent the population, which increases the likelihood that we cannot generalize our findings to a larger group. Thus, future studies should replicate these findings with larger sample sizes. Second, the screening measures used (CSID and AQ) were not yet validated in the SSA/DRC. Third, this study included 2 categories of participants which are suspected dementia and healthy controls. Participants who were seen in between the spectrum (e.g., MCI, subjective memory complaints) were excluded, leaving only the extremes of the dementia spectrum. Future studies should include all the 4 groups (healthy controls, MCI, subjective memory complaint and dementia). Other limitations include lack of normative data for the fluid biomarkers used here in the studied population, relatively small sample size for a biomarker study, and no replication cohort. Cardiovascular risk factors also appeared relatively low in this sample. Future studies should aim to replicate our findings in a larger population by analyzing essential predictors associated with WMHs and Alzheimer’s disease, such as Socioeconomic status (SES) and biomarkers of Alzheimer’s disease (Beta-amyloid, Tau, and APOE4) that were not included in our study. Furthermore, future research should extend these findings by incorporating comprehensive cognitive domain analyses to elucidate the specific cognitive deficits associated with WMHs in the Congolese population. While our study established a link between WMHs and dementia, it remains unclear how different cognitive domains—such as executive function, processing speed, memory, and visuospatial abilities—are differentially affected by WMH burden. Prior studies have demonstrated that WMHs are particularly associated with deficits in executive function and processing speed, given their disruption of fronto-subcortical networks (Vernooij et al., 2009; Prins and Scheltens, 2015). However, given the heterogeneity in cognitive aging and dementia presentations across diverse populations, future studies in SSA should incorporate standardized neuropsychological assessments tailored to cultural and linguistic contexts to ensure accurate characterization of cognitive impairment (Ogunniyi et al., 2006). Additionally, examining the interplay between WMHs and other neuroimaging markers—such as cortical atrophy and microstructural integrity—may provide a more comprehensive understanding of the neural mechanisms underlying cognitive decline in this understudied population. Such research could inform culturally appropriate interventions targeting modifiable risk factors to mitigate cognitive decline associated with WMHs. As this is the first WMHs-related study to be done in DRC, more studies with larger sample sizes are needed in which the association between WMHs and its predictors is examined from the same population for better comparison and further validation of other predictors that were not significantly associated with WMHs in our study, like cardiovascular.

## Data Availability

The raw data supporting the conclusions of this article will be made available by the authors, without undue reservation.
